# Unintentional injury prevention in American Indian and Alaska Native communities: a scoping review of the Indian Health Service Primary Care Provider newsletter

**DOI:** 10.1186/s40621-024-00509-1

**Published:** 2024-06-24

**Authors:** Wendy Shields, Anne Kenney, Evelyn Shiang, Rebecca Malizia, Holly Billie

**Affiliations:** 1 Johns Hopkins Center for Injury Research and Policy, Baltimore, MD USA; 2 Multinational Client Group, Mercer, Baltimore, MD USA; 3 Sala Institute for Child and Family Centered Care, New York University Langone Health, New York, NY USA; 4 Centers for Disease Control and Prevention, Atlanta, GA USA

**Keywords:** American Indian or Alaska Native, Health status disparities, Primary prevention, Public health practice, Wounds and injuries

## Abstract

**Background:**

Unintentional injuries disproportionately impact American Indian and Alaska Native (AI/AN) populations. Developing effective and culturally tailored data collection and intervention programs requires an understanding of past prevention efforts in AI/AN communities, but limited peer-reviewed literature on the topic is available. This scoping review aims to summarize efforts that have been published in the Primary Care Provider newsletter, a source of gray literature available through the Indian Health Service.

**Methods:**

The research team obtained all injury related articles in the Provider newsletter and excluded those that did not describe an unintentional injury prevention effort. Included articles were organized chronologically and by topic, and outcomes were described in a data abstraction form.

**Results:**

A total of 247 articles from the Provider newsletter were screened, and 68 were included in this review. The most number of articles were published in 2007 (*n* = 15). Many focused not specifically on one tribal community but on the AI/AN community as a whole (*n* = 27), while others reported that certain tribes were the focus of study but did not identify tribes by name (*n* = 24). The following is a list of 14 tribal communities explicitly mentioned: Omaha, Cherokee, Ute, Yakama, Chippewa, Apache, Ho-Chunk, The Crow Tribe, Tohono O’odham Nation, Fort Mojave Tribe, Chemehuevi Tribe, The Rosebud Tribe, Navajo, and The Pueblo of Jemez. Published unintentional injury prevention efforts have covered the following 7 topics in AI/AN communities: falls, motor vehicle crashes, poisonings, improving data, burns, children, and other.

**Conclusion:**

This scoping review makes available and searchable information on injury prevention work conducted in and for AI/AN communities that is not currently found in the peer-reviewed literature.

## Background

Unintentional injuries are a worldwide public health problem and disproportiantely impact American Indian and Alaska Native (AI/AN) populations. Unintentional injuries are the leading cause of death for AI/AN people ages 1–44 and the third leading cause of death across all ages (U.S. Department of Health and Human Services. Indian Health Focus: Injuries 2017). Between 2018–2021, the AI/AN age-adjusted unintentional injury death rate (64.0 per 100,000 standard population) was greater than the rate across all races (54.7) (Fatal Injury Data Visualization and [Internet] 2024). There are 573 federally-recognized AI/AN tribes in the United States (US), with 61 additional state-recognized tribes, each with unique environments, culture, and infrastructures. The large number of unique tribes presents challenges on many fronts.

To address unintentional injury disparities, researchers and practitioners have made efforts to develop and tailor surveillance methods and interventions in certain tribal communities (Pollack et al. 2011; Virtue et al. 2024). However, systematic reviews have revealed that peer-reviewed publications are few for AI/AN populations. The topic of AI/AN motor vehicle crash injuries has the greatest number of peer-reviewed publications, yet the number is not commensurate with the magnitude of the problem. Pollack et al. (2011) in “Motor Vehicle Deaths Among AI/AN Populations” stated in their discussion, “The small number of studies in the peer-reviewed literature is surprising given the enormous human and economic impact of motor-vehicle-related deaths in this population.” In a recent systematic review of violent injury prevention efforts, the authors suggested that published studies underrepresent the total range of programs implemented in AI/AN communities due to complexities in tribal infrastructures, such as jurisdictional concerns with data (Rollman et al. 2024).

Improved knowledge of prior unintentional injury prevention activities in Indian country would support program planning to reduce disparities in a manner that is consistent with the injury equity framework (Kendi and Macy 2023). This approach to addressing disparities recognizes injury risk as a product of the intersection between an individual’s identity, historical and current societal contexts, and the following five contributors and countermeasures to injury risk at the family- and community-level: 1) built and natural environment, 2) local and state legislation and policy, 3) education, 4) equipment and technology, and 5) treatment and recovery (Kendi and Macy 2023). Although effective surveillance and evidence-based countermeasures have been developed across many injury problems for the general public, the current literature provides little insight on the unique structural factors that may impact the outcomes of prevention programs in AI/AN communities. Access to findings from past efforts in AI/AN populations can highlight the complex influences driving injury disparities, allowing researchers and practitioners to develop solutions with more effective cultural tailoring and implementation plans.

Although not necessarily peer-reviewed, gray literature can help fill the current knowledge gap by shedding light on injury problems, intervention results, and lessons learned in specific AI/AN communities. An excellent source of gray literature on AI/AN injury prevention is the Indian Health Service (IHS) Primary Care Provider. The Primary Care Provider was a newsletter that published monthly issues from January 1997 to November 2017 (U.S. Department of Health and Human Services 2024). While the newsletter is no longer being published by the IHS, it reached more than 8,000 health care providers working for IHS; tribal and urban Indian health programs; medical, pharmacy, and nursing schools; and other health professionals working with or interested in AI/AN health care. The primary aims of this newsletter were to: a) publish educational articles on topics of clinical interest to IHS and tribal and urban Indian health care professionals; b) share problem solving strategies that might be duplicated elsewhere; and c) apprise readers of current literature relating to AI/AN populations. Importantly, all studies included in the Provider were initiated and published with the permission of all involved tribes. The Provider regularly published articles describing injury prevention projects being conducted through the IHS Injury Prevention Fellowship Program, a training program that included field work. Many of the projects completed by fellowship participants have helped to reduce injuries in AI/AN communities.

Although the Provider is still available on the IHS website as an archive of issues that can be downloaded, the website does not provide a search option for locating specific articles by author or topic. Therefore, the overall purpose of this paper was to catalog and summarize projects addressing unintentional injuries in AI/AN communities that have been published in the IHS Provider newsletter. Given the heterogenous nature of the research included in the Provider, a scoping review approach was used to address the following objective: understand which unintentional injury topics have been addressed in AI/AN populations through studies in the Provider and characterize these studies with attention to observations and outcomes useful for future cultural tailoring and implementation efforts.

## Methods

This scoping review was conducted in accordance with the Preferred Reporting Items for Systematic review and Meta-analyses extension for Scoping Reviews (PRISMA-ScR) Checklist (Tricco et al. 2018). It was not registered and did not have a previously published protocol.

### Eligibility criteria, search strategy, and selection process

The eligibility criteria were developed based on the study objectives. To be included, studies must have met the following criteria: 1) published in the IHS Primary Care Provider newsletter between 1997–2017, 2) described the collection of data related to unintentional injuries or a program aimed at addressing unintentional injuries, and 3) reported data or implemented a program in AI/AN participants specifically. To comprehensively summarize and categorize the projects included in the Provider, this review did not exclude any study or publication type. Studies that met the following criteria were excluded: 1) investigated intentional injuries exclusively, 2) did not report on AI/AN participants, or 3) did not publish in English.

The search for articles was conducted entirely through the IHS website entitled “Primary Care Provider Newsletter: Archive of Issues” (https://www.ihs.gov/provider/archives) (U.S. Department of Health and Human Services 2024). Because this website only displays downloadable issues organized by publication date and therefore does not allow for the search of individual articles, a traditional electronic search strategy could not be completed. Instead, two members of the research team downloaded and reviewed every issue published in the IHS Primary Care Provider newsletter, including special issues focused on injury prevention and specific unintentional injury topics, such as poisonings. No date limits were used.

One member of the research team (RM) screened every article title in the newsletter and excluded all those that did not meet inclusion criteria. Another team member (WS) randomly selected 10% of the newsletter issues (*n* = 25) and checked all included articles to confirm accuracy in screening. Two team members (RM and ES) then independently completed a full-text review of all remaining articles. Any disagreements about eligibility were resolved through discussion with a third member (WS).

### Data collection and synthesis

Each article was read thoroughly by a member of the research team (RM) and summarized in a data abstraction form. The form was developed to address this scoping review’s objectives by capturing a broad range of information relevant for stakeholders interested in program planning to reduce injury disparities. RM recorded the article title; first author; publication date; injury topic (e.g., burns, falls, poisonings); study type (e.g., epidemiologic designs, evaluation types, program description; etc.); and location, as well as a brief project description; the knowledge gap addressed (e.g., local epidemiology, novel intervention, cultural tailoring of an existing intervention); and whether the article contained an evaluation component. If available, RM also recorded the tribe, sample size, summary of findings, and any statistical results. The completed data abstraction forms were verified by another researcher (ES). For ease of presentation, the results were organized, synthesized, and tabulated by injury topic.

## Results

A total of 247 articles were identified from the IHS Primary Care Provider newsletter. Title screening resulted in 97 articles, and full-text review resulted in 68 articles which were included in this scoping review. These articles were published from March 1997 to August 2015. Excluded articles mostly failed to address unintentional injuries at the title screening stage or addressed intentional injuries exclusively at the full-text review stage.

The most number of articles were published in 2007 (*n* = 15). The following 7 topics were covered: falls, motor vehicle crashes, poisonings, improving data, burns, children, and other. Many of the articles did not focus specifically on one tribal community but on the AI/AN community as a whole (*n* = 27). Some articles reported that certain tribes were the focus of the study but did not identify tribes by name (*n* = 24). The following is a list of 14 tribal communities explicitly mentioned: Omaha, Cherokee, Ute, Yakama, Chippewa, Apache, Ho-Chunk, The Crow Tribe, Tohono O’odham Nation, Fort Mojave Tribe, Chemehuevi Tribe, The Rosebud Tribe, Navajo, and The Pueblo of Jemez.

A description of projects and available findings for each included article is provided in Table 1, together with the injury topic, tribe and location, study type, and knowledge gap addressed. Because relatively few studies (*n* = 20) included an evaluation component, and because the study designs and data reported were highly heterogenous even within injury topics, sample sizes and statistical results were not pooled or tabulated separately. If available, statistical results were reported in the individual article’s description of findings in Table 1 and below.

**Table 1:** Summary of articles by injury topic

Topic (Number of articles)	First author (Date published)*	Tribe (Location)	Study type	Knowledge gap addressed	Project description and available findings
Falls (*n* = 15)	Kuklinski (May 1998) (Kuklinski 1998)	Multiple unidentified (Arizona)	Cross-sectional	Local epidemiology, risk identification	Project: Described causes of Phoenix Area elder deaths Finding: The mortality rate due to falls was higher than the rate for all United States (US) races
	Sandstorm (October 1998) (Sandstrom et al. 1998)	Omaha (Nebraska)	Cross-sectional	Local epidemiology	Project: Employed the Physical Therapy Assessment and Treatment Protocol at a skilled nursing facility Finding: The rate of functional impairments was high among residents
	Maxted (November 1998) (Maxted 1998)	Multiple unidentified (Not available [N/A])	Tool development	Cultural tailoring of an existing measurement tool	Project: Adapted a functional assessment to American Indian and Alaska Native (AI/AN) elders Finding: A composite measure was likely to be the most accurate means of assessing functionality in elders
	McDonald (February 2001) (McDonald 2001)	AI/AN generally (N/A)	Protocol	Local epidemiology, risk identification	Project: Developed a community-based participatory research approach to collecting survey data on the health and social needs of AI/AN elders
	Finke (May 2003) (Finke 2003)	AI/AN generally (N/A)	Qualitative analysis	Cultural tailoring of an existing intervention	Project: Sought feedback from clinicians to adapt preventative care guidelines for elders to AI/AN populations Finding: Recommendations were collected on screening, immunizations, chemoprophylaxis, and more
	Morse (May 2005) (Morse 2005)	AI/AN generally (N/A)	Literature review	Cultural tailoring of existing interventions	Project: Described the use of health promotion programs in primary care settings to improve the health of elders Findings: Community-based health promotion programs in Indian Health Service (IHS) primary care settings have particularly benefited AI/AN elders
	Ducore (July 2008) (Ducore and Newsad 2008)	Multiple unidentified (California)	Tool development	Novel measurement tool	Project: Developed the Elder Falls Prevention Self-Assessment Tool to capture fall injury risk factors Finding: Test clinics reported that the tool addressed their need for a fall prevention assessment but could be clarified for easier use
	Bill (July 2010) (Bill and Finke 2010)	AI/AN generally (N/A)	Protocol	Cultural tailoring of existing interventions	Project: Created workgroups to identify data, evidence-based interventions, and existing AI/AN efforts as part of a comprehensive approach to elderly fall prevention
	Michaelson-Gambrell (July 2010) (Michaelson-Gambrell and Williams 2010)	One unidentified (N/A)	Implementation evaluation	Cultural tailoring of an existing intervention	Project: Developed and implemented a Tai Chi program to reduce elderly falls Finding: Community members’ initial responses were receptive
	Berger (October 2010) (Berger and Sims 2010)	Multiple unidentified (N/A)	Cross-sectional	AI/AN-specific epidemiology	Project: Quantified the extent of polypharmacy among AI/AN elders Finding: In 2009, 73% of patients aged > 50 received ≥ 1 prescription, 43% received ≥ 4 prescriptions, 24% received ≥ 7 prescriptions, and 13% received ≥ 10 prescriptions during at least one medical encounter
	Sims (July 2011) (Sims et al. 2011)	Multiple unidentified (N/A)	Cross-sectional	AI/AN-specific epidemiology	Project: Quantified prescriptions associated with increased fall injury risk among AI/AN elders Finding: In 2008, 19% of patients aged ≥ 65 received a prescription associated with increased fall injury risk
	Scott (July 2013) (Scott et al. 2013)	One unidentified (British Columbia)	Implementation and outcome evaluation	Novel intervention	Project: Evaluated the effectiveness and reception of a multimedia training program to increase fall prevention knowledge in primary care providers Finding: After the program, participants’ knowledge of elderly falls increased
	Stevens (September 2013) (Stevens 2013)	AI/AN generally (N/A)	Program description	Novel intervention	Project: Developed a tool kit to act as a broad resource to help health care providers incorporate fall risk assessment and interventions into clinical practice
	Finke (July 2013) (Finke and Bill 2013)	AI/AN generally (N/A)	Literature review	Cultural tailoring of existing interventions	Project: Summarized guidelines for fall prevention in AI/AN elders Finding: A comprehensive fall prevention approach should include screening, exercises to improve mobility, and solutions to medications with side effects
	Berger (July 2013) (Berger and Williams 2013)	AI/AN generally (N/A)	Program description	Cultural tailoring of existing interventions	Project: Created strategies to incorporate fall prevention into the comprehensive care of adults with diabetes
Motor vehicle crashes (MVCs) (*n* = 14)	Parris (July 2010) (Parris 2010)	One unidentified (N/A)	Qualitative analysis	Cultural tailoring of existing interventions	Project: Interviewed multiple stakeholders to understand the keys to sustaining a child passenger safety program in tribal communities Finding: Key elements included child restraint law, advocacy, and resources
	Letourneau (July 2011) (Letourneau et al. 2011)	Four unidentified (N/A)	Qualitative analysis	Cultural tailoring of existing interventions	Project: Determined contributors and barriers to success in tribal motor vehicle and injury prevention programs (TMVIPPs) Finding: Success in TMVIPPs were due to program administration, partnerships, tailoring strategies, and more
	Hansen (August 2015) (Hansen and Hymer 2015)	One unidentified (Nevada, Arizona, and New Mexico)	Program description	Novel intervention	Project: Improved an existing training program called Safe Native American Passengers to increase participation with the goal of reducing MVC injuries in children
	Phipps (March 1997) (Phipps et al. 1997)	Cherokee (Oklahoma)	Cross-sectional	Local epidemiology	Project: Quantified the cost and rate of MVC-related emergency room visits and hospitalizations at an IHS hospital Finding: Between January and September 1994, there were 262 MVC-related patients, most of whom were not restrained, accounting for about $506,000
	Williams (June 1998) (Williams 1998)	Ute (Utah)	Implementation and outcome evaluation	Cultural tailoring of an existing intervention	Project: Created an incentive campaign to increase car seat and seat belt use by rewarding drivers and passengers for being properly restrained Finding: Post-incentive observations showed that rates at least doubled in both types of restraint use
	John (November 2001) (John and Berger 2001)	Yakama (Washington)	Program description	Cultural tailoring of existing interventions	Project: Reviewed multiple interventions including a child restraint education and distribution program, a survey to assess seat belt use, a public awareness campaign, and efforts to pass a seat belt law
	Thompson (September 2003) (Thompson et al. 2003)	Chippewa (Minnesota)	Tool development	Cultural tailoring of an existing measurement tool	Project: Used geographic information system (GIS) technology to develop a map of motor vehicle crash site clusters
	Reede (July 2007) (Reede et al. 2007)	Apache (Arizona)	Outcome evaluation	Cultural tailoring of existing interventions	Project: Increased sobriety checkpoints, lowered the legal blood alcohol content limit, and created a media campaign to increase restraint use and reduce alcohol-impaired driving Finding: Between 2004–2006, MVCs involving injury reduced by 20%
	Billie (September 2007) (Billie et al. 2007)	Ute (Utah)	Outcome evaluation	Cultural tailoring of existing interventions	Project: Implemented general and targeted education events, enhanced law enforcement, and child safety seat clinics to increase restraint use Finding: Between 2002–2003 and 2004–2005, adult and child restraint use grew from 22 to 42% and 20% to 42% respectively, and alcohol-related MVCs declined
	Letourneau (July 2009) (Letourneau 2009)	Ho-Chunk (Wisconsin)	Outcome evaluation	Cultural tailoring of existing interventions	Project: Implemented general and targeted education events, media campaigns, and enhanced law enforcement to increase restraint use Finding: Between 2005–2008, seatbelt use increased from 46.7% to 61.2%, and between fall 2003 and spring 2008, child safety seat use increased from 26.4% to 78.4%
	Piland (December 2010) (Piland et al. 2010)	Apache (Arizona)	Cost–benefit evaluation	Cultural tailoring of existing interventions	Project: Compared the costs and benefits of a comprehensive TMVIPP Finding: For every dollar spent over eight years, there were almost $10 in medical and other cost savings
	Tsatoke (February 2010) (Tsatoke et al. 2010)	One unidentified (N/A)	Outcome evaluation	Local epidemiology	Project: Explained the statistical decline in MVC injuries at an IHS emergency room Finding: The reduction was due to 76% of MVC-related patients being transported to other facilities
	Merchant (June 2012) (Merchant 2012)	Crow (Montana)	Cross-sectional	Local epidemiology	Project: Identified locations where MVCs occurred using transportation data and GIS technology Finding: Between 2002–2008, there were 36 MVC cluster sites, and annual MVC rates were lower than statewide averages but with higher severity indices
	Smith (July 2014) (Smith et al. 2014)	Six unidentified (Northwestern region)	Protocol	Novel intervention	Project: Developed methods for using an observational interview to measure child safety seat use, including site and vehicle selection
Poisonings (*n* = 9)	Dreisbach (December 2006) (Dreisbach and Koester 2006)	Seven unidentified (Colorado)	Qualitative analysis	AI/AN-specific epidemiology	Project: Conducted interviews with current and former methamphetamine users and service providers to describe the trends, signs, and implications of use Finding: Collaborative prevention, early detection, and treatment efforts could reduce risk in AI/AN communities
	Bubar (December 2006) (Bubar and Payne 2006)	One unidentified (N/A)	Literature review	AI/AN-specific epidemiology	Project: Summarized the impact of methamphetamine use on children Finding: Higher incidences of child abuse and neglect were related to methamphetamine use
	Honahni (December 2006) (Honahni 2006)	Multiple unidentified (N/A)	Cross-sectional	Risk identification	Project: Surveyed law enforcement agencies to identify the threat level of methamphetamine on tribal lands Finding: The most reported themes were related to supply and production, community impact and resources, law enforcement challenges, and drug courts
	Hagen (December 2006) (Hagen and Chaney 2006)	AI/AN generally (N/A)	Literature review	Cultural tailoring of existing interventions	Project: Outlined a basic legal framework for the federal prosecution of crimes in AI/AN communities dealing with illegal drugs
	Woodis (January 2007) (Woodis 2007)	AI/AN generally (N/A)	Program description	Cultural tailoring of existing interventions	Project: Summarized the development of a workgroup between the IHS and Substance Abuse and Mental Health Services Administration to address methamphetamine prevention
	Masis (January 2007) (Masis 2007)	AI/AN generally (N/A)	Program description	Cultural tailoring of existing interventions	Project: Developed a model for intervening with methamphetamine users who are willing to quit called the “5 A’s”: Ask, Advise, Assess, Assist, and Arrange
	Lovell (January 2007) (Lovell 2007)	AI/AN generally (N/A)	Program description	Cultural tailoring of existing interventions	Project: Described how Healing to Wellness Courts have supported recovery for substance abusing offenders while improving service delivery and achieving maximum use of resources
	Coyhis (January 2007) (Coyhis and Simonelli 2007)	Multiple unidentified (Colorado)	Qualitative analysis	Cultural tailoring of existing interventions	Project: Summarized discussions at a conference on methamphetamine healing Finding: Participants most often cited the importance of existing knowledge on the drug, cultural tailoring, sharing personal successes, and spiritual approaches
	Love (January 2007) (Love and Barrera 2007)	AI/AN generally (N/A)	Literature review	Cultural tailoring of existing interventions	Project: Documented the issues of and evidence-based interventions for methamphetamine use Finding: Key risk factors for have been identified, but few evidence-based interventions are available in AI/AN populations
Improving data (*n* = 9)	Griffith (July 2002) (Griffith 2002)	AI/AN generally (N/A)	Program description	Local and AI/AN-specific epidemiology	Project: Described a hypothetical conversation between two colleagues planning a Resource and Patient Management System (RPMS) population-based analysis to guide such work
	Powers (November 2006) (Powers 2006)	AI/AN generally (N/A)	Program description	Local and AI/AN-specific epidemiology	Project: Outlined training programs focused on the use and implementation of Electronic Health Records
	Pahona (October 2007) (Pahona et al. 2007)	Multiple unidentified (Nevada, Utah, and California)	Implementation evaluation	Local epidemiology	Project: Tested the viability of using a RPMS to surveil severe injuries Finding: The RPMS provided a reliable and practical process for identifying injury events and types
	Price (July 2008) (Price et al. 2008)	Multiple unidentified (California)	Formative evaluation	Local epidemiology	Project: Interviewed and surveyed injury prevention coordinators on their needs related to storing, retrieving, and comparing passenger safety data Finding: The web-based Occupant Protection Use System (OPUS) addressed coordinators’ common data analysis challenges
	Bradley (January 2009) (Bradley and Nail-Chiwetalu 2009)	AI/AN generally (N/A)	Program description	AI/AN-specific epidemiology	Project: Described the Native Health Database, a web-based system containing citations, abstracts, and full-text links on AI/AN health information
	Piontkowski (February 2011) (Piontkowski et al. 2011)	Multiple unidentified (Arizona)	Outcome evaluation	Local and AI/AN-specific epidemiology	Project: Compared data from local IHS Severe Injury Surveillance Systems and the Arizona Department of Health Services Finding: State data underrepresented injury cases, showing that they cannot completely substitute local data
	Bowser (July 2012) (Bowser and Williams 2012)	Tohono O’odham Nation (Arizona)	Implementation evaluation	Cultural tailoring of an existing intervention	Project: Piloted a Global Positioning System program to enhance the emergency response to MVCs Finding: The response time for combined ambulance and fire truck runs was 16.8 min during baseline and 13.9 min during intervention
	Bales (July 2012) (Bales et al. 2012)	Fort Mojave and Chemehuevi (California, Nevada, and Arizona)	Implementation evaluation	Local epidemiology	Project: Tested an injury surveillance system based on emergency room, hospitalization, and mortality data from multiple states Finding: Through the multi-state system, the leading causes of injuries could be identified
	Dankovchik (July 2014) (Dankovchik et al. 2014)	Multiple unidentified (Washington)	Outcome evaluation	Local epidemiology	Project: Evaluated the accuracy of race information in the Washington Trauma Registry Finding: Nearly half of the AI/AN patient files in the Registry were misclassified by race
Burns (*n* = 3)	Kuklinski (September 1999) (Kuklinski 1999)	Multiple unidentified (Arizona)	Protocol	Cultural tailoring of existing interventions	Project: Developed the Sleep Safe program to reduce fire and burn injuries in children aged 0–5 by providing curricula around smoke detector use and emergency planning
	Kuklinski (July 2007) (Kuklinski and Cully 2007)	Multiple unidentified (Minnesota)	Implementation and outcome evaluation	Cultural tailoring of existing interventions	Project: Implemented the Sleep Safe program in 20 of the 27 Bemidji Area tribes with Head Start programs Finding: Across all sites, the number of homes with functioning smoke alarms increased 40 percent (from 70% at baseline to 99% at follow-up visits)
	Kuklinski (May 2001) (Kuklinski and Allen 2001)	Chippewa (Minnesota)	Program description	Cultural tailoring of existing interventions	Project: Created the White Earth Home Safety Collaborative Team to educate elders on how to maintain and test smoke alarms
Children (*n* = 1)	Berger (July 2007) (Berger et al. 2007a)	AI/AN generally (N/A)	Cross-sectional	AI/AN-specific epidemiology and risk identification	Project: Described injury mortality rates in AI/AN children and children in the US overall between 2000–2002 Finding: The disparity in mortality rates due to injury was the main driver of the disparity in overall mortality rates between AI/AN and White children
Other (*n* = 17)	Russell (March 2001) (Russell et al. 2001)	Rosebud (South Dakota)	Cross-sectional	Local epidemiology and risk identification	Project: Characterized animal bite cases in an emergency room Finding: 396 cases were identified, suggesting the following needs: a revised hospital animal bite protocol, increased community awareness, and a Dog Control Task Force
	Brown (October 2004) (Brown and Finke 2004)	AI/AN generally (N/A)	Literature review	Cultural tailoring of existing interventions	Project: Summarized the risk factors for and approaches to screening osteoporosis Finding: Common screening guidelines were particularly appropriate for elderly AI/AN women
	Cooper (October 2004) (Cooper 2004)	AI/AN generally (N/A)	Literature review	AI/AN-specific epidemiology	Project: Described how to use the Cochrane Database for indexing systematic reviews of medical, surgical, and health interventions
	Carlson (June 2005) (Carlson et al. 2005)	AI/AN generally (N/A)	Implementation evaluation	Cultural tailoring of an existing intervention	Project: Assessed the response of IHS injury practitioners to a free online injury training course Finding: The option resulted in high rates of nonparticipation
	Rothman (July 2005) (Rothman 2005)	Navajo (Arizona)	Cross-sectional	Local epidemiology	Project: Surveyed children on their all-terrain vehicle use and safety practices Finding: Although 73.6% of students grades 5–8 reported riding an ATV at least once per week, only 67% of those students reported at least usually wearing a helmet
	Riddles (November 2006) (Riddles 2006)	AI/AN generally (N/A)	Outcome evaluation	Cultural tailoring of existing interventions	Project: Reviewed the performance of IHS agencies and programs based on government-wide measures Finding: Between 2002–2006, the US Office of Management and Budget reviewed 6 IHS programs, all rated narratively as Adequate or higher
	Berger (February 2007) (Berger et al. 2007b)	AI/AN generally (N/A)	Outcome evaluation	Novel intervention	Project: Surveyed IHS Injury Prevention Fellow graduates to understand whether the program achieved its goals Finding: Three or more years after program completion, 48% of graduates said that injury prevention constituted at least 25% of their current workload
	Letourneau (July 2007) (Letourneau and Crump 2007)	AI/AN generally (N/A)	Program description	Cultural tailoring of existing interventions	Project: Described benefits and best practices for the IHS’s provision of technical assistance to enhance injury prevention capacity in tribes
	Robertson-Begay (September 2007) (Robertson-Begay et al. 2007)	Navajo (Arizona)	Program description	Cultural tailoring of existing interventions	Project: Increased injury prevention capacity through a multipronged approach, including assessment of key issues and the creation of the Hardrock Council on Substance Abuse Prevention
	Piland (September 2007) (Piland and Berger 2007)	AI/AN generally (N/A)	Literature review	AI/AN-specific epidemiology	Project: Reviewed information about the economic burden of injuries among AI/AN communities Finding: The total cost of injuries was over $2.1 billion in 2000
	Hicks (September 2007) (Hicks et al. 2007)	AI/AN generally (N/A)	Program description	Cultural tailoring of existing interventions	Project: Created the IHS Injury Prevention Program’s guiding principles, including community-specific interventions, collection of reliable data, and capacity building to foster tribal ownership
	Prosser (July 2008) (Prosser and All 2008)	Multiple unidentified (California)	Qualitative analysis	Novel intervention	Project: Conducted interviews of practitioners to understand the role of Contract Health Services (CHS) in AI/AN health care services Finding: Data on injuries were scarce because summaries from CHS hospitals were not required prior to payment by the local clinic
	Tsatoke (July 2009) (Tsatoke et al. 2009)	One unidentified (N/A)	Program description	Novel intervention	Project: Described the benefits and challenges of establishing injury prevention partnerships in one tribe, such as funding issues and turnover
	Berger (July 2011) (Berger and Piontkowski 2011)	AI/AN generally (N/A)	Program description	Cultural tailoring of an existing intervention	Project: Described the advantages of using focus groups in community-based injury interventions, such as flexibility, affordability, and rapid feedback
	Berger (July 2012) (Berger 2012)	AI/AN generally (N/A)	Program description	Cultural tailoring of an existing intervention	Project: Described beneficial approaches to community engagement for injury prevention, such as sourcing insights from community members, partnerships, and targeted use of media
	Canniff (July 2014) (Benton 2015)	Multiple unidentified (Oregon)	Implementation evaluation	Cultural tailoring of existing interventions	Project: Documented the challenges faced by a set of tribes in creating a full-time injury prevention program Finding: Challenges included lack of awareness of the impact of injury, funding, and difficulty accessing data
	Benton (August 2015) (Benton 2015)	Pueblo of Jemez (New Mexico)	Implementation evaluation	Cultural tailoring of existing interventions	Project: Documented the successful establishment of a full-time injury prevention program Finding: Factors that contributed to the successful implementation included communication with tribal leadership, partnerships with tribal and non-tribal entities, and evidence-based strategies

*Refer to the references list to determine the full article’s issue and page number range

### Falls

The most number of articles covered fall-related injury prevention in AI/AN communities (*n* = 15), though only 13% (*n* = 2) included an evaluation component. Fall-related articles focused generally, though not exclusively, on AI/AN elders. An article published in May 1998 examined the causes of 282 AI/AN elder deaths over a 15-year period in the Phoenix Area and offered guidance for elder injury prevention. The total injury mortality rate for Phoenix Area elders was more than double the rate for all US races (Kuklinski 1998). An article published on the Omaha Tribe found that restorative care needs were not being met at a Nebraska nursing home using the Physical Therapy Assessment and Treatment Protocol. The researchers looked at locomotion, transfers, and bed mobility to determine the functional status of participants (Sandstrom et al. 1998). A later study described the process for clinicians to accurately diagnose and treat patients, deciding that a composite measure is likely to be the most accurate means of assessing functionality of elders (Maxted 1998). A February 2001 study reported the health and social needs of AI/AN elders compared to the general US. population to be used for grant writing efforts, statistical evidence needs, and lobbying of state legislature (McDonald 2001).

In May 2003, the IHS Provider released age-specific, evidence based preventative care guidelines for elders to be updated periodically. The guidelines outline practical preventive care for AI/AN elders including screening, immunizations, chemoprophylaxis, counseling, diet and exercise, injury prevention, dental health, and sexual behavior (Finke 2003). In May 2005, an article described how a community-based program can secure health benefits for elders and support the role of the IHS in the community to increase physical and psychosocial health (Morse 2005).

In July 2008, three health clinics in California released an “Elder Falls Prevention Self-Assessment Tool”. The evaluation components included risk factor screening, medication review, gait and balance, muscle weakness, reduced physical fitness, behavioral risk factors, environmental risk factors, history of falls, visual acuity and depth perception, and documentation (Ducore and Newsad 2008). In July 2010, the IHS reported a plan to develop a comprehensive approach to the prevention of fall-related activities in elders living in the community by creating workgroups that addressed the need for data, evidence, and inventory (Bill and Finke 2010). Another report described the positive response of community members to the institution of a Tai Chi program that could reduce falls among older adults in AI/AN communities. Tai Chi classes were offered in several communities and the researchers produced both a book and a DVD about Tai Chi movements and benefits (Michaelson-Gambrell and Williams 2010).

One important factor that contributes to falls in AI/AN elders is polypharmacy. One article identified the extent of polypharmacy among AI/AN elders using the IHS National Data Warehouse for FY 2009. The researchers found that 73% of patients aged above 50 received at least one prescription, 43% received four or more prescriptions, 24% received seven or more prescriptions, and 13% received ten or more prescriptions during at least one medical encounter (Berger and Sims 2010). A later study found that although use of specific medications and multiple medications potentially increase the risk of falls in elders, these occurred frequently among AI/AN elders (Sims et al. 2011). In British Columbia, a tribe developed, evaluated, and disseminated a user-friendly, evidence-based fall prevention multimedia training program for primary care providers who interact often with elders who have experienced a fall (Scott et al. 2013). Additionally, the Stop Elderly Accidents, Deaths, and Injuries tool kit was developed to act as a broad, evidence-based resource to help health care providers incorporate fall risk assessment and individualized fall interventions into clinical practice (Stevens 2013). Another article published guidelines for fall prevention in AI/AN elders, including education about falls and risk factors; exercises that improve mobility, strength and balance; a review of medications with side effects or drug interactions that contribute to falls; vision exams by trained healthcare professionals; and home safety assessment and home modification (Finke and Bill 2013). Only one article addressed the increased risk of fall injuries among AI/AN individuals with diabetes, providing strategies for incorporating fall prevention activities into the comprehensive care of adults with diabetes (Berger and Williams 2013).

### Motor vehicle crashes (MVCs)

A total of 14 articles focused on motor vehicle crash prevention. Compared to articles covering other injury topics, MVC-related articles had the second-highest proportion of evaluation studies, with 43% (*n* = 6) involving an evaluation component. In 2010, an unidentified tribe conducted a literature review, informant interviews, and focus groups to identify key elements for sustaining a child passenger safety program. These elements included: advocacy, child restraint law, resources, partnerships, policies guiding implementation, accessible services, data and evaluation, and program coordination (Parris 2010). In 2011, an evaluation of tribal motor vehicle and injury prevention programs (TMVIPPs) funded by the Centers for Disease Control and Prevention in four tribes determined that the success of these programs was due to four major components: program administration, partnerships/collaboration, tailoring effective strategies within AI communities, and data collection and evaluation (Letourneau et al. 2011). In 2015, an unidentified tribe located in Nevada, Arizona, and New Mexico improved an existing training program called Safe Native American Passengers to increase participation with the ultimate goal of reducing motor vehicle injuries in children (Hansen and Hymer 2015).

The Cherokee Tribe in Tahlequah, Oklahoma evaluated the cost-effectiveness of motor vehicle injury prevention programs by determining the cost of hospitalization and emergency room visits due to MVCs at a hospital in 1997. The researchers concluded that increasing the rate of seat belt and child restraint usage can dramatically reduce serious injuries and medical costs from MVCs (Phipps et al. 1997). In 1998, the Ute Tribe created an incentive campaign to increase car seat and seat belt use on the Uintah and Ouray Reservation in Fort Duchesne, Utah by observing drivers and passenger restraint usage and rewarding them for being properly restrained (Williams 1998). In 2001, the Yakama Nation in South Yakima, Washington conducted a review of different interventions including a child restraint education and distribution program, a survey to assess seat belt use, a public awareness campaign, and efforts to pass a primary seat belt law (John and Berger 2001). In 2003, the Chippewa on Fond du Lac Reservation in Northeastern Minnesota evaluated specific motor vehicle injuries by utilizing geographic information system (GIS) technology to develop a map of motor vehicle crash site clusters on the reservation. The study found two major crash cluster sites (Thompson et al. 2003).

In 2007, the Apache on the San Carlos Apache Reservation in Arizona increased sobriety checkpoints, lowered the legal blood alcohol content (BAC) limit to 0.8%, and created a media campaign to increase restraint use and reduce alcohol-impaired driving (Reede et al. 2007). The Ute tribe on the Uintah and Ouray Reservation in northern Utah successfully increased adult and child restraint use and reduced both alcohol related and unimpaired crashes through the implementation of evidence-based strategies. Adult restraint use increased from 22 to 42%; child restraint use increased from 20 to 42%. The total number of alcohol-related crashes declined from 32 to 21 (34%) between 2002–2003 and 2004–2005 (Billie et al. 2007).

In 2009, the Ho-Chunk Nation in Wisconsin implemented general education events, targeted education, media awareness, training, enforcement, and policy to increase child restraint use and seat belt use (Letourneau 2009). In 2010, the Apache Tribe studied the economic cost and injury outcomes of the TMVIPP in San Carlos, Arizona (Piland et al. 2010). The study found that for every dollar spent to implement the prevention program, there were almost $10 in savings from medical and other costs. Another project in 2010 sought to explain a reduction in annual MVC injuries despite no change in tribal laws to increase preventative measures. The researchers concluded that 76% of the 139 MVC-related patients were taken to other facilities, bypassing triage at the local IHS hospital (Tsatoke et al. 2010). In 2012, the Crow Tribe evaluated the frequency and severity of MVCs on the Crow Indian Reservation in Montana by identifying locations where crashes occurred using transportation data and GIS technology (Merchant 2012). In 2014, six tribes in the Northwest observed vehicles and interviewed drivers of children, determining that more children were properly restrained in 2011 and 2013 than they had been in 2009 (Smith et al. 2014).

### Poisonings

A total of 9 articles covered the prevention of poisonings, all of which specifically focused on drug use, but none included an evaluation component. Several articles from December 2006 focused on reducing methamphetamine use among tribal members. Seven rural communities in Colorado described the trends, signs, and implications of methamphetamine use in rural settings and by AI/AN communities by conducting interviews with current and former methamphetamine users and service providers (Dreisbach and Koester 2006). Another tribe determined that higher incidences of child abuse and neglect in tribal communities were related to methamphetamine use (Bubar and Payne 2006). To inform policy decisions, develop stronger interagency partnerships, provide more drug enforcement training, and engage in education and prevention efforts, researchers surveyed 96 Indian country law enforcement agencies to identify the threat level of methamphetamine production and use on tribal lands and the impact on tribal communities and law enforcement agencies (Honahni 2006). Finally, another article provided a basic legal framework used in the federal prosecution of crimes in AI/AN communities by clarifying that federal drug offenses are applicable and allows for felony federal prosecution of persons dealing with methamphetamine, or other illegal drugs, within Indian country (Hagen and Chaney 2006).

The IHS and Substance Abuse and Mental Health Services Administration’s Methamphetamine Initiative Workgroup collaborated with universities, task forces, outpatient centers, and communities to address substance abuse prevention, specifically methamphetamine prevention (Woodis 2007). One model recommended for intervening with tobacco users who are willing to quit is the “5 A’s”: Ask, Advise, Assess, Assist, and Arrange (Masis 2007). Healing to Wellness Courts, a response to the judicial and treatment systems’ failure to effectively address substance abuse and its related activity, focus on healing of individuals, families, and communities. These courts have paved pathways to recovery for substance abusing offenders and their families while also improving service delivery and achieving maximum use of limited resources (Lovell 2007). A conference convened by White Bison, Inc. in April 2006 in Denver, Colorado discussed existing knowledge about methamphetamine, successes in AI/AN communities, select personal healing and recovery success stories, and a vision for future healing practices (Coyhis and Simonelli 2007). Another article identified issues that commonly lead to methamphetamine use such as abuse of other substances, mental and physical health problems, and barriers to a generally healthy lifestyle (Love and Barrera 2007).

## Improving data

Several articles focused on improved collection of data as a tool to improve unintentional injury prevention in AI/AN communities (*n* = 9). These articles had the highest proportion of evaluation studies compared to other injury topics, with 67% (*n* = 6) of articles including an evaluation component. In one article, a hypothetical conversation was published between two fictitious colleagues planning a Resource and Patient Management System (RPMS) population-based analysis to guide preparation for such work (Griffith 2002). One article described the IHS’s use of Electronic Health Records and discussed the steps of implementation and lessons learned (Powers 2006). Another described efforts by the Reno District IHS Office of Environmental Health and Engineering Division of Environmental Health Services to develop a consistent, reliable, and practical process for identifying the number and types of severe injury at each of the communities using RPMS (Pahona et al. 2007).

The Occupant Protection Use System (OPUS), a web-based occupant protection data collection and warehouse system with security features, encourages wider participation by AI/AN communities in obtaining usage data to allow comparison among reservations and with state and national rates. OPUS allows for real time storage and retrieval of survey results that consist of data entry, reports, administration, and resources (Price et al. 2008). Meanwhile, the Native Health Database, a web-based resource used to find health and medical information on AI/AN populations, contains citations, abstracts and full-text links to about 8,500 documents, eliminating the need to search across multiple databases and websites on AI/AN health or medical information (Bradley and Nail-Chiwetalu 2009). To determine the plausibility of relying solely on state injury prevention data and avoid the time, effort, and cost involved in local data collection, researchers examined cases identified in existing IHS Severe Injury Surveillance Systems (SISS). No fatalities and 58 injury hospitalizations were identified by the SISS, yet 51 injury hospitalizations and 5 fatalities were identified by the Arizona Department of Health Services and Vital Statistics Section data (Piontkowski et al. 2011).

One article evaluated a pilot program that used a Global Positioning System to reduce response time of local law enforcement for the Pisinemo District of the Tohono O’odham Nation in Arizona. The response time for the combined ambulance and fire truck runs during the baseline period was 16.8 min and 13.9 min during the intervention period (Bowser and Williams 2012). In the same month, researchers worked to build an injury surveillance system based on emergency room, hospitalization, and mortality databases for the Fort Mojave Tribe and Chemehuevi Tribe. Of the 540 reported injuries, the six underlying causes were falls, struck by/against, overexertion, cut/pierce, MVC, and assault (Bales et al. 2012). In the Portland area, a study evaluated the completeness and accuracy of race information within the Washington Trauma Registry. Results showed that nearly half of the AI/AN cases in the registry were misclassified. Before correcting for this race misclassification, the data underestimated AI/AN injury rates and the scope of disparities experienced by the AI/AN population in Washington (Dankovchik et al. 2014).

### Burns

The prevention of burn injuries was covered in 3 articles, one (33%) of which included an evaluation component. The “Sleep Safe” fire safety program sought to reduce fire and burn injuries in AI/AN children ages 0–5 by providing curricula to teachers, providers, children, and tribal partnerships that emphasized the use of smoke detectors and the development of an action plan if a fire does start (Kuklinski 1999). Several tribes in the Bemidji Area participated in the “Sleep Safe” program, provided education on fire safety, and installed smoke alarms to reduce residential fire-related mortality of AI/AN children. “Sleep Safe” saw success because it provided 10-year batteries and photoelectric alarms, educated parents about smoke alarm importance, and promoted parental involvement in community fire safety efforts (Kuklinski and Cully 2007). The Chippewa at White Earth Reservation in Minnesota also created the White Earth Home Safety Collaborative Team to educate residents on how to maintain and test smoke alarms but did not complete a follow-up evaluation to determine its effectiveness (Kuklinski and Allen 2001).

### Children

Only 1 article focused on unintentional injury prevention in children generally, and it was not an evaluation. This study examined disparities in child mortality rates among AI/AN children and youth compared to the overall child mortality rate in the US. For AI/AN children ages 1–19 years, the number of injury deaths is far greater than the number of deaths from the next seven leading causes combined (1,040 vs. 182; ratio of 5:7:1); for White children, the number of injury deaths also outnumbers the next seven leading causes (35,961 vs. 11,339; 3:2:1). For children ages 1–4 and 15–19 years, the percentage of deaths due to unintentional injuries was similar for both AI/AN and White children. Additionally, homicide caused a large proportion of the deaths to young AI/AN children (Berger et al. 2007a).

### Other

The remaining 17 unintentional injury prevention articles describe a wide range of topics, with 29% (*n* = 5) including an evaluation component. One study investigated dog bite related injuries on the Rosebud Reservation in South Dakota. The study was conducted to epidemiologically characterize dog bite injuries, with emphasis on the evaluation of medical treatment lag time and investigation lag time. The study decided that the following recommendations should be implemented: review and revise hospital animal bite protocol, raise community awareness, establish a Dog Control Task Force, establish a database of non-fatal dog bite injuries, and continue the coordination of spaying and neutering clinics (Russell et al. 2001).

One study outlined guidelines to approach osteoporosis and fracture prevention in the IHS. It offered strategies for applying US Preventive Services Task Force recommendations to AI/AN populations and suggests an evidence-based public health approach to fracture prevention in a health system with limited access and resources (Brown and Finke 2004). Another article described how to use the Cochrane Database for indexing systematic reviews of medical and surgical therapies and other health care interventions (Cooper 2004). Yet another investigated online training as a continuing education option for IHS injury prevention staff. Of the 68 injury professionals who were invited to participate in a free online course, only two registered for the workshop; one completed the pretest; and neither completed the posttest (Carlson et al. 2005).

In July 2005, the Provider published a study conducted on all-terrain vehicle (ATV) injuries of children of the Navajo Tribe in Kayenta, Arizona. Children under 16 years accounted for 37% of injuries and 33% of all deaths from ATVs. Of students who owned an ATV, only 28.6% of high school students reported owning a helmet and 31.5% of students grades 5–8 owned a helmet (Rothman 2005).

The IHS Performance Achievement Team monitored performance management and found that the IHS Sanitation Facilities Construction Program, the RPMS, Health Care Facilities Construction Program, and the Federally Administered Health Program were rated moderately effective; the Urban Indian and Tribally-Operated Health Programs were rated adequate (Riddles 2006). A long-term evaluation of the IHS Injury Prevention Fellowship Program sought to determine the effectiveness of the program through criteria measures three or more years after completion of the training. Of the 86 respondents, 48% said that injury prevention constituted at least 25% of their current workload; 71% said that injury prevention constituted at least 5% of their current workload (Berger et al. 2007b). One article described the context and components of technical assistance provided to the Tribal Injury Prevention Cooperative Agreements Program and their implications for other federal IHS programs. The article concluded that technical assistance was more likely to be effective if based on a long-standing relationship of trust, particularly in AI/AN communities that have had significant negative experiences with outside experts entering the community and making assumptions (Letourneau and Crump 2007).

The Navajo Tribe of Hardrock assessed the key issues contributing to injury related deaths and created the Hardrock Council on Substance Abuse Prevention. They also developed programs to increase seat belt use and smoke alarm use, and fostered support and involvement of local leaders to promote sustained funding (Robertson-Begay et al. 2007). In the year 2000, researchers found the direct and indirect costs from injuries to AI/AN communities were over $2.1 billion. These costs included: economic burden, Years of Potential Life Lost, cost of medical treatment, law enforcement costs, and others. For AI/AN, 40% of the Years of Potential Life Lost before age 65 is due to injuries (Piland and Berger 2007). The Provider also published the IHS Injury Prevention Program’s guiding principles: community-specific interventions, collection of reliable data, capacity building to foster tribal ownership of the programs, and partnership building to collaborate to increase efficiency and effectiveness (Hicks et al. 2007).

A public health nurse and outreach director of the Feather River Tribal Health Clinic recounted an attempt to develop an injury prevention program targeting frequent and serious injuries among AI/AN populations. They found that data on injuries were scarce since discharge summaries from Contract Health Services (CHS) hospitals and clinical summaries from CHS referral providers were not required prior to payment by the clinic. They concluded that data collection and accuracy can be improved through improved coding and requiring standardized processes for health care workers (Prosser and All 2008).

A case study of injury prevention partnerships in Indian country studied the effect of collaboration on injury prevention in an AI tribe. Tribes face difficulties when forming injury prevention partnerships due to historical tensions, interpersonal conflicts, and disputes about funding allocation. However, these partnerships have proven beneficial in reducing injuries in Indian country (Tsatoke et al. 2009). Another report demonstrated how focus groups are advantageous to facilitate community-based services and interventions for several reasons, including: in-depth feedback, perceptions, attitudes, beliefs, and feelings of the target audience are revealed due to the flexible format; a moderator can ask probing questions if further clarification is necessary; more expansive thinking of participants is promoted by the interactive nature; and they are relatively inexpensive, require minimal resources, and provide rapid feedback (Berger and Piontkowski 2011). Community engagement is helpful in preventing injuries in AI/AN communities. Approaches to engage the community include gaining insights from community members, formation of partnerships, and targeted use of media (Berger 2012).

Some tribes discuss challenges faced when creating a full-time injury prevention program. The Northwest Portland Area Indian Health Board cited the following difficulties: lack of awareness of the impact of injury, underfunding, and difficulty finding access to high-quality, relevant local data on injury (Canniff et al. 2014). The Pueblo of Jemez in New Mexico seemed to overcome these difficulties and created an evidence-based injury prevention program with an extensive network of partners. The Pueblo of Jemez credit their success to communication with tribal leadership, partnerships with tribal and non-tribal entities, evidence-based strategies, reliable data, and respect for traditional customs and language (Benton 2015).

## Discussion

Using the IHS Primary Care Provider, this scoping review identified 7 unintentional injury topics that have been addressed in AI/AN populations. Many of the reviewed articles covered knowledge gaps in local epidemiology and the cultural tailoring of existing interventions. Compared to articles in the peer-reviewed literature, articles in the Provider included a wider range of publication types, including multiple kinds of evaluation (formative, implementation, outcome, and cost–benefit), protocols, and descriptions of programs designed for specific AI/AN communities. Because all studies published by the Provider were initiated, conducted, and approved through partnerships with tribal councils, they provided unique insights into the social and administrative environments of tribal communities. For example, multiple articles discussed how structural factors influenced the challenges and benefits of past prevention efforts in AI/AN populations, which would be useful for researchers and practitioners aiming to develop solutions to injury disparities.

Although the research published through the Provider would be valuable to many stakeholders in the fields of tribal health and injury prevention, several barriers explain the persisting scarcity of such work in the peer-reviewed literature. First, studies done in tribal communities often require permission from tribal councils to be conducted and published. In addition to administrative and jurisdictional complexities, a history of exploitation by the scientific community has generated hesitation to participate. In this review, many articles did not identify specific tribe names even when permission was granted to complete the study. Additionally, those leading injury programs in AI/AN communities have largely been practitioners with limited capacity to engage in the academic publishing process. As a source of gray literature, the Provider was able to accept work that may have been more difficult to publish in peer-reviewed journals, such as analyses with inconclusive results or brief program descriptions.

### Limitations

This scoping review should be interpreted in the context of the following limitations. First, the accuracy of our conclusions was limited by the quality of the articles included. Although all articles published in the Provider underwent an approval and selection process supervised by editors at the IHS, not all articles in the newsletter were necessarily peer-reviewed. As a result, the studies in this review may not reflect an exact substitution for peer-reviewed literature. Furthermore, the most recent publication date included in this review was in 2015. Given that the Provider was discontinued after 2017, this review could not characterize more recent AI/AN prevention activities. Our scoping review approach was also associated with some limitations. While it was our goal to categorize the wide heterogeneity of study designs and publication types in the Provider, this heterogeneity made it difficult to conduct critical appraisals and draw statistical conclusions from the studies.

## Conclusion

This article provides a summary of the breadth and depth of the unintentional injury prevention work that has been conducted and published through the IHS Primary Care Provider newsletter. Others aiming to do injury prevention work with AI/AN communities can use this article as a reference for previous work done in the field. This publication also highlights the need for more peer-reviewed documentation of injury prevention projects conducted in AI/AN populations. Collaborative solutions involving researchers, practitioners, and AI/AN communities to address the barriers to publishing in Indian country would support the injury prevention field’s increasing focus on reducing disparities.

## Data Availability

The articles analyzed in the current review are available at the Primary Care Provider Newsletter: Archive of Issues website: https://www.ihs.gov/provider/archives/

## Abbreviations

AI/AN: American Indian and Alaska Native
US: United States
HIS: Indian Health Service
TMVIPPs: Tribal motor vehicle and injury prevention programs
GIS: Geographic information system
BAC: Blood alcohol content
RPMS: Resource and Patient Management System
OPUS: Occupant Protection Use System
SISS: Severe Injury Surveillance Systems
ATV: All-terrain vehicle
CHS: Contract Health Services
